# A Rapid and Accurate Bioluminescence-Based Migration Assay Permitting Analysis of Tumor Cell/Stromal Cell Interactions

**DOI:** 10.3390/mps3010010

**Published:** 2020-01-23

**Authors:** Jinsoo Yoon, Christopher R. Parish, Lucy A. Coupland

**Affiliations:** The ACRF Department of Cancer Biology & Therapeutics, John Curtin School of Medical Research, The Australian National University, Canberra 2601, Australia; Jinsoo.Yoon@anu.edu.au (J.Y.); Christopher.Parish@anu.edu.au (C.R.P.)

**Keywords:** transwell migration assay, tumor/stromal interactions, bioluminescence

## Abstract

Bioluminescent tumor cell lines are used extensively in vivo to monitor tumor growth and metastasis but rarely used in vitro to follow tumor cell behavior. Tumor cell migration is frequently studied in vitro using transwell assays, however, current methods do not permit the co-incubation of tumor cells with different stromal cell types for analysis of the effects of intercellular cross-talk on tumor cell migration. We describe a novel migration assay using bioluminescent tumor cell lines that is rapid, accurate, and permits the study of the effects of tumor cell-stromal cell interactions on tumor cell migratory behavior.

## 1. Introduction

**Figure 1:**
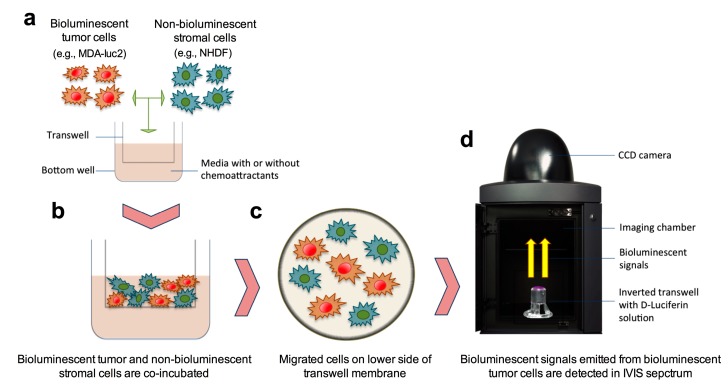
Schematic illustration of the co-incubation of bioluminescent tumor cells and non-bioluminescent stromal cells in the bioluminescence migration assay. (**a**) Bioluminescent tumor cells and non-bioluminescent stromal cells were seeded and (**b**) incubated together in transwells. (**c**) Both bioluminescent tumor and non-bioluminescent stromal cells are likely to have migrated through the membrane. (**d**) Following removal of the nonmigrated cells from the upper side of the membrane, the bioluminescent signal from the migrated bioluminescent tumor cells on the lower side of the membrane are detected by the IVIS® Spectrum, an in-vivo imaging system, after the addition of D-luciferin solution.

The development of bioluminescent tumor cell lines and low-light imaging chambers has greatly increased the capacity for research into factors influencing tumor cell growth and migration in vivo. The potential for these systems to study tumor cell behavior in vitro, however, has not been realized. Most often, in vitro tumor cell migration studies employ the transwell migration assay (also known as the modified Boyden chamber assay or filter assay) that was initially developed by Boyden to analyze the chemotactic responses of leukocytes [1]. The principle of this assay is that cells, seeded in the upper chamber, migrate through a porous membrane into the lower chamber, with or without the stimulation of a chemo-attractant. Migrated cells are then stained and quantified either by a microscopic technique, wherein migrated cells are counted in a number of representative fields, or by colori- or fluoro-metric assays following cell lysis [2]. The transwell migration assay is well established and frequently used in the field of cell migration research including that of tumor cell metastasis. One of the major drawbacks of the current staining and quantification protocols, however, is the limitation to the use of one cell type. As the influence of cell signalling, particularly from stromal cells, on tumor cell migration is being increasingly recognized [3], we established a transwell migration assay wherein stromal cells can be co-incubated with bioluminescent tumor cells and the effect of this co-incubation on the bioluminescent tumor cells is quantified using low-light imaging facilities (Figure 1). Furthermore, this newly established bioluminescent assay can be performed more rapidly and with substantially increased accuracy.

## 2. Experimental Design

The aim of these experiments was to determine the migratory capacity of bioluminescent tumor cell lines following direct interaction with platelets or fibroblasts. The standard in vitro transwell migration assay was used with the measurement of migrated cells determined using a novel bioluminescent method described herein, as well as standard crystal violet staining.

In the first experiment, the human osteosarcoma cell line, 143B, that had been transfected with retroviral particles containing the luciferase gene, was seeded within the transwells at varying densities and the number of migrated cells was determined 24 h later using the novel bioluminescent method and compared with the crystal violet staining method.

In the second experiment, the bioluminescent murine breast cancer cell line, 4T1-luc2, was directly co-incubated with or without platelets (1 tumor cell:1000 platelets) within the transwell and the impact upon cell migration was determined 24 h later using the novel bioluminescent method and compared with the crystal violet staining method.

In the final experiment, the bioluminescent human breast cancer cell line, MDA-MB-231-luc2, was directly co-incubated with or without the non-bioluminescent fibroblast cell line, NHDF, in a 1:1 ratio for a period of 24 h, then the extent of migration was determined using the novel bioluminescent method and compared with the crystal violet staining method.

To briefly summarize the novel bioluminescence method described in greater detail below, in each of these experiments, nonmigrated tumor cells that remained on the interior transwell membrane were removed, the transwells were placed within the IVIS^®^ Spectrum imaging chamber with the exterior membrane surface facing upwards, D-luciferin solution was added to the membrane surface, and the bioluminescence images were acquired using the IVIS^®^ Spectrum in vivo imaging system and Living Image^®^ 4.4 software. The number of migrated cells per transwell was then calculated based upon previously determined luminescence/cell values.

### 2.1. Materials

#### 2.1.1. Cell Lines

- Murine mammary gland carcinoma (4T1-luc2) (PerkinElmer, Waltham, MA, USA; Cat. No.: 124087)
- Human breast carcinoma MDA-MB-231-luc2 (MDA-luc2) (PerkinElmer; Waltham, MA, USA; Cat. No.: 124319)
- Human osteosarcoma (143B) (American Type Culture Collection; ATCC, Manassas, VA, USA; Cat. No.: CRL-8303)
- Human dermal fibroblast (NHDF) (Lonza, Basel, Switzerland; Cat. No.: CC-2511).

The complete growth medium and culture conditions for each cell line should be obtained from the datasheet provided by the supplier.

#### 2.1.2. Reagents

- RPMI 1640 medium for 4T1-luc2 (Thermo Fisher Scientific, Waltham, MA, USA; Cat. No.: 21870-076)
- Minimum essential medium (MEM) for MDA-luc2 and 143B (Sigma-Aldrich, St. Louis, MO, USA; Cat. No.: M5650)
- Dulbecco’s modified eagle medium (DMEM) for NHDF (Sigma-Aldrich; Cat. No.: D5671)
- Fetal bovine serum (FBS) (GE Healthcare Bio-Sciences, Pittsburgh, PA, USA; Cat. No.: SH30071.03)
- 10X Trypsin-EDTA (Thermo Fisher Scientific; Cat. no.: 15400-054)
- Penicillin-streptomycin-neomycin (PSN) (Thermo Fisher Scientific; Cat. No.: 15640-055)
- Phosphate-buffered saline (PBS) (Sigma-Aldrich; Cat. no.: 806544)
- RediFect red-fluc-puromycin lentiviral particles (PerkinElmer; Waltham, MA, USA; Cat. no.: CLS960002)
- Puromycin (Invivogen, Pak Shek Kok, Hong Kong; Cat. No.: Ant-pr-1)
- Hexadimethrine bromide (polybrene) (Sigma-Aldrich; Cat. no.: TR-1003-G)
- D-luciferin (PerkinElmer; Cat. No.: 122796)
- Crystal violet solution (0.1% crystal violet in 2% ethanol, Sigma-Aldrich; Cat. No.: V5265)
- Nunc^TM^ EasYFlask^TM^ cell culture flask in 25 and 75 cm^2^ (Thermo Fisher Scientific; Cat. No.: 156367 and 156499)
- Costar^®^ 24-well clear TC-treated multiple well plates (Corning, New York, NY, USA; Cat. No.: 3524)
- 6.5 mm Transwell^®^ with 8.0 μm pore polycarbonate membrane insert (Corning; Cat. No.: 3422)
- Sterile cotton swabs

### 2.2. Equipment

- CO_2_ incubator at 37 °C with 95% humidity and 5% CO_2_ (Sanyo Electric, Osaka, Japan; Cat. No.: MCO-20AIC)
- IVIS^®^ Spectrum in vivo imaging system (PerkinElmer; Cat. No.: IVISSPE)
- Inverted Bright-field/fluorescence microscopy (Olympus Life Science, Center Valley, PE, USA; Cat. No.: IX71)

### 2.3. Software

- Living Image^®^ 4.4 software (PerkinElmer, Waltham, MA, USA; Cat. No.: 128113)
- Image-Pro^®^ Plus (Media Cybernetics, Rockville, MD, USA)
- GraphPad Prism 6 (GraphPad Software, San Diego, CA, USA)

## 3. Procedure for the Co-Incubation of Bioluminescent and Non-Bioluminescent Cell Lines

### 3.1. Insertion of Cells into Transwells (Time for Completion: 20–30 min Not Including Incubation Period)

- Prepare separately luciferase-transduced and nonluciferase-transduced cells (1.25–2.5 × 10^5^ cells/mL of each cell line) in media.
- Add 600 μL of media to the lower well of the 24-transwell plate.
- Add 100 μL of each of the cell suspension solution (luciferase-transduced cell and nonluciferase-transduced cell, total 200 μL) into the interior of the transwell.
- Gently insert the transwell into the lower well and incubate the plate for 6–24 h in a cell culture incubator.

**NOTE:** If bubbles are present under the transwell membrane, remove the air bubbles with a sterile pipette tip, as they will inhibit cell migration, then gently re-insert the transwell and incubate.

### 3.2. Measurement of Migrated Cells

#### 3.2.1. Initiation of IVIS^®^ Spectrum and Living Image^®^ software

- Start ‘Living Image^®^ 4.4 software’
- Click ‘Initialize’ on the IVIS acquisition control panel to start up the system and raise the temperature (Figure 3 No. 1).
- Click ‘Imaging Wizard’ on the control panel (Figure 3 No. 2).
- Select ‘Bioluminescence’ and ‘Next’ in the imaging mode (Figure 3 No. 3–4).
- Select ‘Open Filter’ and ‘Next’ in the imaging option (Figure 3 No. 5–6).
- Select ‘Well Plate’ in the imaging subject and ‘Next’ (Figure 3 No. 7–9).

#### 3.2.2. Transwell Washing and Removal of Nonmigrated Cells (Time for Completion: 3–4 Min)

**Figure 2:**
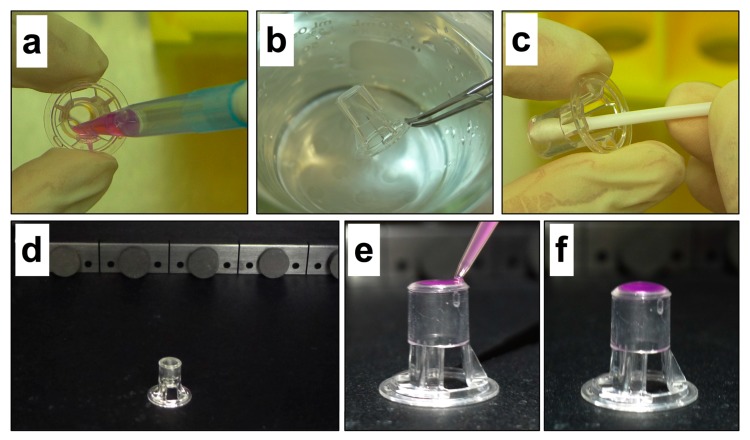
The processing steps of the bioluminescence migration assay. (**a**) Removal of nonmigrated cells and medium from the interior of the transwell. (**b**) Washing transwell in phosphate buffered saline (PBS). (**c**) Removal of nonmigrated cells and residual moisture from the interior of the transwell using a cotton swab. (**d**) Transwells are placed in the imaging chamber of the IVIS^®^ Spectrum in an inverted position and (**e**) 40 μL of D-luciferin solution gently pipetted onto the exterior membrane surface. (**f**) Transwell is ready for bioluminescence imaging.

- Aspirate and discard the media containing nonmigrated cells from the inside of the transwell (Figure 2a).
- Wash the transwell twice in PBS (Figure 2b).
- Gently swab the interior of the transwell twice using water-soaked cotton swabs to remove nonmigrated cells attached to the interior of the transwell membrane (Figure 2c).
- Swab the interior of the transwell twice using dry cotton swabs to remove any residual moisture (Figure 2c).

**NOTE** Take care not to puncture the membrane or touch the exterior surface of the transwell membrane.

#### 3.2.3. Imaging the Transwells for Bioluminescence (Time for Completion: 1–2 min)

**Figure A1:**
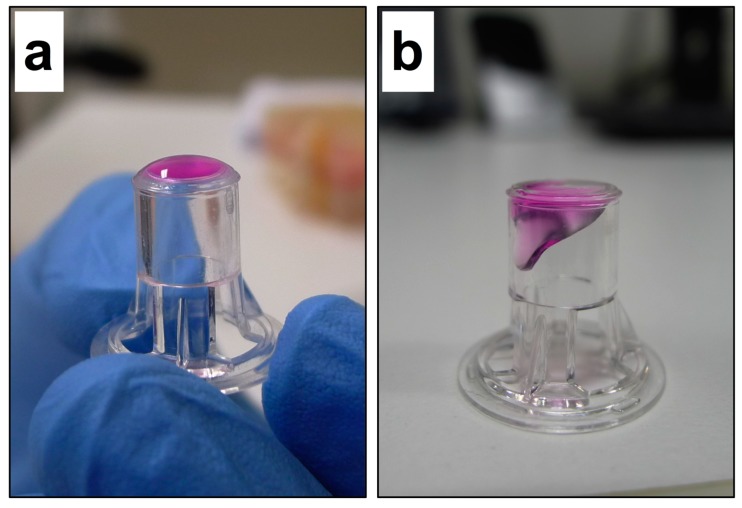
Correct and incorrect placement of D-luciferin solution on inverted transwells. (**a**) The D-luciferin solution should remain above the membrane and not flow through as seen in (**b**). Thorough removal of excess moisture from the inside of the transwell will prevent this from occurring.

- Invert and place the transwell on the base of the IVIS^®^ Spectrum imaging chamber (Figure 2d).

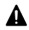
**CRITICAL STEP** Make sure the transwell is placed within the imaging chamber as soon as possible after cleaning to prevent migrated cells from drying out.

ii. Gently pipette 40 μL D-luciferin solution upon the exterior membrane of the transwell (Figure 2e).

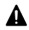
**CRITICAL STEP** Ensure the transwell membrane is completely covered by the D-luciferin solution (Figure 2f and Figure A1a).

**NOTE** Do not touch the surface of the membrane with the pipette tip to avoid dislodging migrated cells on the membrane surface.

**Figure 3:**
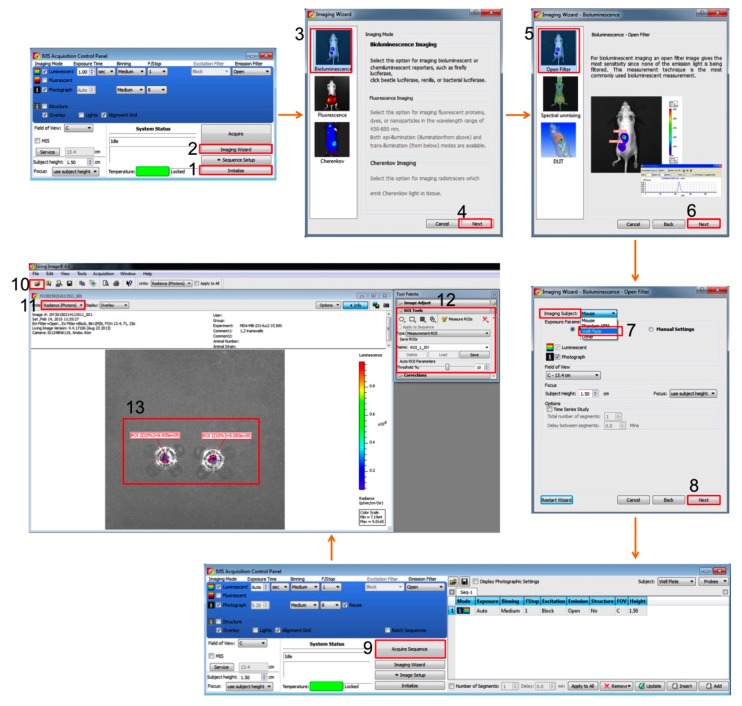
Flowchart of software procedure containing screen shots of key steps. Equipment setup is in no. 1–8 with red rectangles. Measurement and data analysis are in no. 9–12 with red rectangles. No. 13 is an example of bioluminescent signal from transwells.

**NOTE** Gently close the imaging chamber door to minimize disturbance of the D-luciferin solution (Figure A1b).

iii. Click on “Acquire” in the control panel (Figure 3 No. 9).

**NOTE** According to the manufacturer’s (Perkin Elmer; Waltham, MA, USA) instructions, the time period when cells are incubated with the D-luciferin solution should be brief at 37 °C before the image is obtained. Based upon the work of ‘Shinde et al. (2006) [4]’, the kinetics of the bioluminescent signal from cells in vitro following the addition of D-luciferin show an initial peak at less than 2 min before reaching a plateau then a gradual decline in the signal intensity occurs. The images obtained for the purpose of the studies presented in this manuscript were captured at 1 min post D-luciferin addition. As the signal intensity gradually declines, consistency in timing between the addition of D-luciferin and image capture should be ensured between wells within an experiment. It is possible to image up to three transwells at a time as long as the D-luciferin solution is added to the top of the three transwells within 5 to 10 s and the image is obtained at 60 s.

### 3.3. Analyzing the Images to Quantify Migrated Cells (Time for Completion: 3–4 Min)

- Open the imaging file in the Living Image^®^ 4.4 software and select ‘Radiance (Photons)’ in the units box (Figure 3 No. 10–11).
- Click and open ‘ROI Tools’ in the tool palette (Figure 3 No. 12).
- Click the 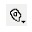 button next to ‘Measurement ROIs’ and select ‘Auto All’ on the drop-down list (Figure 3 No. 12).

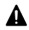
**CRITICAL STEP** The software automatically draws measurement regions of interest (ROIs) on all images. The ROI label shows the total intensity within the ROI and the threshold %.

iv. Use the threshold % slider and click ‘Measure ROIs’ button in the ROI tools to show the ROI measurements table (Figure 3 No. 12–13).

## 4. Expected Results

**Figure 4:**
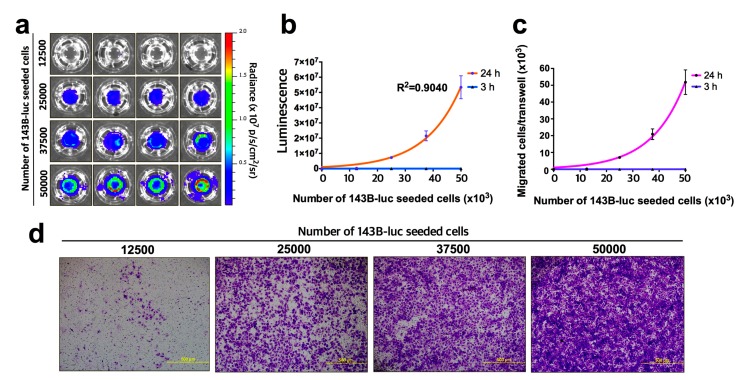
Seeded tumor cell number correlates highly with migrated tumor cell number as measured using bioluminescence imaging. (**a**) 143B-luc osteosarcoma cells were seeded in transwells (n = 4) at the cell densities indicated, incubated for 24 h and then the migrated tumor cells imaged after the addition of D-luciferin using the IVIS^®^ Spectrum. (**b**) Intensity of the luminescent signal received from transwells versus the number of seeded tumor cells at 3 and 24 h. (**c**) Luminescence data in (**b**) converted to the number of migrated tumor cells per transwell (value for luminescence/cell obtained from standard curve—Figure A2). (**d**) Representative images of crystal violet staining of migrated tumor cells at 24 h at tumor cell seeding densities as indicated (100× magnification). Bars represent SEM.

**Figure A2:**
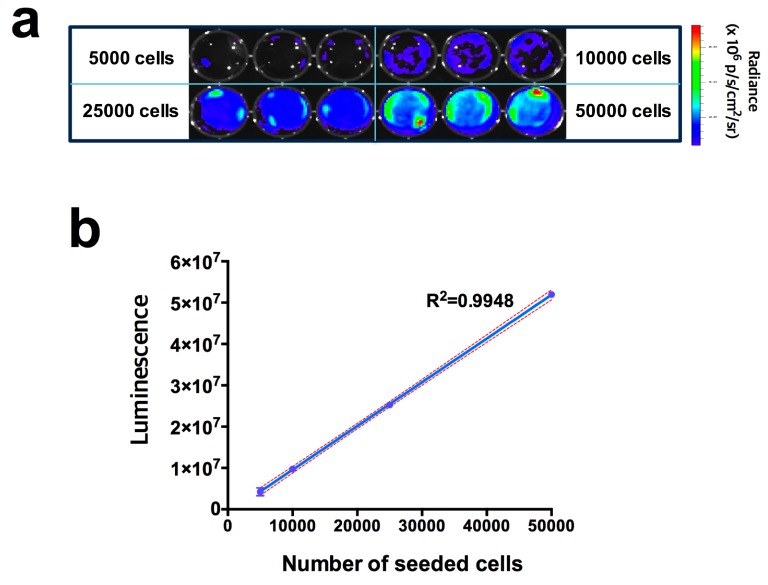
Generation of a standard curve of bioluminescence. (**a**) 143B-luc cells were added to wells of plates at the cell numbers were indicated, D-luciferin was added, and the bioluminescent signal obtained was quantified using the IVIS spectrum. (**b**) Graph of bioluminescent signal versus number of seeded cells. Bars represent SEM (R^2^ = 0.9948).

Initially, to demonstrate the accuracy of bioluminescent quantification of migrated tumor cells, human osteosarcoma cells transduced with RediFect red-fluc-puromycin lentiviral particles (143B-luc) were seeded at 12.5, 25, 37.5, and 50 × 10^3^ cells per transwell (n = 4) with a medium in the upper and lower transwell chambers and incubated for 3 and 24 h at 37 °C in 5% CO_2_. The transwells were processed and the bioluminescent intensity of migrated tumor cells were determined as briefly described above and in detail in the procedure (Figure 2). The bioluminescent signal of migrated tumor cells correlated highly with the number of seeded cells (R^2^ = 0.9040) (Figure 4b). These data were converted to the number of migrated tumor cells/transwell (Figure 4c) through the inclusion of a standard curve (Figure A2). A similar migration curve result was obtained with the human breast carcinoma cell line, MDA-MB-231-luc2 (MDA-luc2) (data not shown). Following bioluminescence imaging, the transwells were then stained with crystal violet and examined under a light microscope. Images for the migrated tumor cells at 24 h for all cell seeding densities are shown and demonstrate the difficulty in determining the number of migrating tumor cells using this method, particularly at high cell densities, and hence, the inability to generate an accurate tumor cell migration curve (Figure 4d).

**Figure 5:**
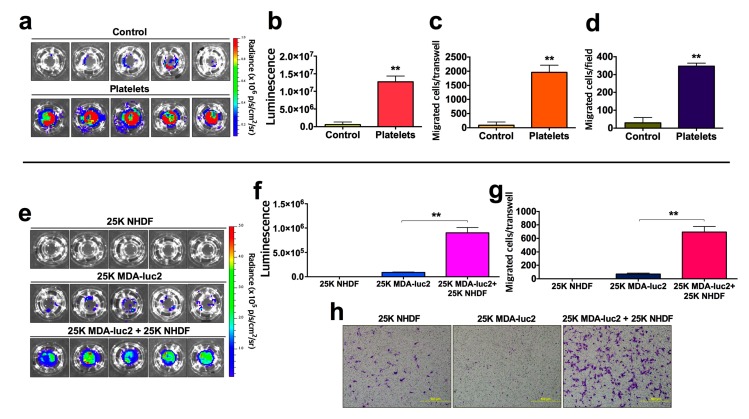
Comparison of bioluminescence and classical migration assays in tumor cell:platelet and tumor cell:stromal cell interaction experiments. (**a**) 4T1-luc2 mammary adenocarcinoma cells were incubated in transwells for 24 h with (n = 5) or without (n = 5) isolated mouse platelets, then the migrated tumor cells imaged using the bioluminescence method. (**b**) Bar graph of luminescent signal obtained from (**a**). (**c**) Conversion of data in (**b**) to number of migrated tumor cells per transwell based upon previously determined luminescence/cell values. (**d**) Number of migrated tumor cells per transwell were determined using crystal violet staining and quantification by Image-Pro Plus from three random microscopic fields. (**e**–**g**) MDA-luc2 tumor cells and NHDF cells (fibroblasts) were incubated separately and together (n = 5) for a 24 h period, then the number of migrated MDA-luc2 tumor cells per transwell determined using the bioluminescence method. (**h**) Migrated cells per transwell were imaged following crystal violet staining using light microscopy (100× magnification), with tumor cells. As one cell type is indistinguishable from the other using this method, a count of migrated tumor cells could not be obtained. Bars represent SEM. ** *p* < 0.01 (unpaired *t*-test).

To compare the bioluminescence method with a standard method of quantification of migrated tumor cells, we performed a transwell migration assay wherein the bioluminescent murine mammary adenocarcinoma cell line, 4T1-luc2, was incubated with or without platelets for a 24 h period then, the number of migrated tumor cells were determined, firstly, using the bioluminescence method then, secondly, staining the same transwells with crystal violet solution, obtaining images of three representative microscopic fields per transwell and using Image-Pro Plus software to calculate the number of migrated tumor cells/field (refer to ‘5.3. **Crystal violet staining**’). The presence of platelets significantly enhanced the migratory potential of the 4T1-luc2 cells (*p* < 0.01) with a 22-fold increase in migrated tumor cells being measured via the bioluminescence method (Figure 5a–c) and a 12-fold difference measured via the crystal violet method (Figure 5d). These data illustrate the similarity in the qualitative results obtained between the two methods, however, the quantification of migrated cells between the two methods differs significantly. As the effect of platelet-treatment on tumor cell migration is dramatic, the crystal violet method would require significant optimization to reduce the density of seeded cells in order to render the migrated cell number sufficiently low to enable accurate counts to be obtained. However, it can also be seen (Figure 5a) that the pattern of cell migration across the membrane is uneven and, consequently, when examining the crystal violet stained membrane under the microscope, it becomes difficult to distinguish one cell from another to obtain an accurate cell count. Hence, the inclusion of the whole transwell surface in the bioluminescent quantification process is more accurate than a selection of representative microscope fields in the crystal violet quantification method.

Finally, to illustrate the ability of the bioluminescence migration assay to facilitate the study of tumor cell/stromal cell interaction effects on tumor cell migration (Figure 1), 25 × 10^3^ luciferase-transduced MDA-luc2 breast carcinoma cells and 25 × 10^3^ nontransduced NHDF were incubated separately or together in transwells (n = 5) for a 24 h period prior to processing and quantification using the bioluminescence and crystal violet methods. No bioluminescent signal was detected from the migrated nontransduced NHDF, a low number of bioluminescent MDA-luc2 tumor cells migrated when seeded alone, however, a significantly (*p* < 0.01) higher number of MDA-luc2 tumor cells migrated when they were co-incubated with NHDF (Figure 5e–g). Crystal violet staining of the transwells showed a similar increase in the number of migrated cells when the two cell lines were co-incubated, however, it was not possible to distinguish between the two cell types for quantification purposes and, therefore, to ascertain the influence of stromal cells on the migration of tumor cells (Figure 5h).

The major advantages of the bioluminescence migration assay over the classical methods include the ability to (i) use two or more cell types within a transwell, (ii) image and quantify bioluminescent tumor cells that have migrated without the need for staining and lysis steps, (iii) minimize the loss of migrated tumor cells on the transwell surface through the elimination of cell fixation, cell staining and cell detachment steps, thus increasing accuracy and reproducibility, (iv) image and quantify the whole transwell surface in one step thus removing the inherent error of averaging counts from representative microscopic fields, (v) further decrease the timing of the assay by imaging three transwells at a time, and (vi) ability to subsequently undertake traditional quantification methods on the same transwell if required (Table A1). To aid users of this assay a trouble-shooting guide is presented in Table A2, with correct placement of the D-luciferin substrate in the inverted transwell depicted in Figure A1a.

The potential information gained from studying the effects of tumor cell/stromal cell interactions on tumor cell migration using the in vitro bioluminescent migration assay is considerable. Bioluminescent tumor cell lines may be co-incubated with one or more other cell types in the upper chamber to determine the influence of direct contact between tumor cells and other normal (stromal) cells upon tumor cell migration (Figure 1). Alternatively, a bioluminescent tumor cell line may be incubated in the upper chamber with one or more normal (stromal) cell types in the lower chamber, thus determining the influence of soluble factors on tumor cell migration. This assay could also be used in the research areas of leukocyte migration and developmental biology.

As the IVIS^®^ Spectrum imaging system has the capacity to measure bioluminescence and fluorescence, it would be possible to also apply this methodology to fluorescent and auto-bioluminescent cell lines. It would be important, however, to predetermine the reliability of the relationship between the fluorescent/auto-bioluminescent signal and cell number to ensure that accuracy is achieved in the calculation of the number of migrated cells. If using fluorescent or auto-bioluminescent cell lines, all steps would be the same except that the addition of D-luciferin (step ii of Section 3.2.3) would not be required but it is recommended that media is added to the upper membrane in the same manner to maintain optimum health of the cells throughout the imaging process. In the setup of the IVIS^®^ Spectrum system it would also be important to select fluorescence or bioluminescence, as appropriate.

Currently used transwell migration assays contain multiple processing steps as summarized in Table A1. In contrast, the in vitro bioluminescence migration assay described herein and depicted in Figure 2 involves (i) removal of nonmigrated cells from the upper transwell chamber and any moisture using cotton swabs, (ii) inverting and placing up to three transwells in the imaging chamber, (iii) placing a D-luciferin solution on the top of the transwells, and (iv) immediately obtaining the image. If the number of migrated cells is required the bioluminescent intensity/cell can be calculated through the inclusion of a titration of cells to generate a standard curve of the bioluminescent signal (Figure A2). To aid users of this assay a trouble-shooting guide is presented in Table A2, with correct placement of the D-luciferin substrate in the inverted transwell depicted in Figure A1.

To conclude, the bioluminescence method for quantifying cell migration requires fewer processing steps than microscope or plate reader-based assays and provides a count of migrated cells across the whole transwell, hence, is more rapid and accurate. The greatest benefit, however, is that cells may be co-incubated in the transwell and the influence of one cell type on the migration behavior of the other can be easily determined.

## 5. Reagents Setup

### 5.1. Establishment of Luciferase-Transduced 143B Cells

25 × 10^3^ 143B tumor cells were seeded in complete MEM media in a 24-well plate. After 24 h, the medium was replaced with a fresh medium containing 30 MOIs (multiplicity of infection) of RediFect red-fluc-puromycin lentiviral particles and 4 μg/mL hexadimethrine bromide (polybrene), and then incubated for an additional 24 h. The medium containing lentiviral particles was then replaced with fresh medium and incubated for 24 h. The cells were split into a new 24-well plate with complete MEM medium containing 0.6 μg/mL puromycin to select the transduced cells. The selected-puromycin resistant cells were incubated for seven days with a medium containing puromycin and designated as 143B-luc.

### 5.2. D-Luciferin Solution

A 200× D-luciferin stock solution (30 mg/mL) was prepared in aliquots and frozen at –20 °C for future use. Just prior to use, thaw the 200× stock solution of D-luciferin and dilute 1:200 in a prewarmed complete medium (150 μg/mL final concentration).

### 5.3. Crystal Violet Staining

The transwells were prepared as described in ‘3.1. **Insertion of Cells into Transwells**’ and ‘3.2.2. **Transwell Washing and Removal of Non-Migrated Cells**’. The transwells were then fixed in 4% formaldehyde for 15 min, washed again twice in PBS, and stained in crystal violet solution for 20 min. The transwells were washed three times in distilled water, placed under a light microscope (100× magnification) and three random images collected across the transwell membrane. The number of migrated cells was determined using the Image-Pro Plus software.

## Appendix A

**Table A1:** Comparison of bioluminescence versus classical migration assays.

Processing Steps	Bioluminescence	Cell Count (Classical)	Colorimetry or Fluorometry
Wash	✔	✔	✔
Transwell Cleaning	✔	✔	✔
Cell Staining	✕	✔	✔
Cell Detachment	✕	✕	✔
Cell Lysis	✕	✕	✔
Cell Count	✕	✔	✕
Quantification Steps			
Data Collection and Analysis	Low light imaging system and software	Light microscope with image software	Plate reader and software
Detection Reagent	Firefly luciferase with D-luciferin	Crystal violot Hematoxylin Toluidin blue	Crystal violot or Calcein AM
Assay Time	**Less than 10 min**	**Approximately 1 h**	**Approximately 1 h**

**Table A2:** Troubleshooting guide for bioluminescence migration assay.

Problem	Possible reason	Solution
Absence of a bioluminescent signal	- Problem with D-luciferin solution - No cells have migrated.	- Check bioluminescent efficiency of the luciferase-transduced cells and concentration of D-luciferin working solution. - Stain transwells with crystal violet and check migrated cells using microscope. - If there are no migrated cells, verify your cells have migratory capacity. - When inserting transwells into bottom well ensure no air is trapped between medium and lower side of transwell.
D-luciferin solution leaks down inside of transwell (Figure A1)	- Residual moisture in interior of transwell - Vibration on closing of imaging chamber door	- Repeat transwell washing with PBS and remove moisture inside transwell using dry cotton swab - Close the chamber door carefully
